# Dorsal root ganglia hypertrophy as *in vivo* correlate of oxaliplatin-induced polyneuropathy

**DOI:** 10.1371/journal.pone.0183845

**Published:** 2017-08-24

**Authors:** Leonidas Apostolidis, Daniel Schwarz, Annie Xia, Markus Weiler, Andreas Heckel, Tim Godel, Sabine Heiland, Heinz-Peter Schlemmer, Dirk Jäger, Martin Bendszus, Philipp Bäumer

**Affiliations:** 1 Department of Medical Oncology, National Center for Tumor Diseases, Heidelberg, Germany; 2 Department of Neuroradiology, Heidelberg University Hospital, Heidelberg, Germany; 3 Department of Neurology, Heidelberg University Hospital, Heidelberg, Germany; 4 Department of Radiology, German Cancer Research Center, Heidelberg, Germany; Medical University Innsbruck, AUSTRIA

## Abstract

**Purpose:**

To investigate *in vivo* morphological and functional correlates of oxaliplatin-induced peripheral neuropathy (OXA-PNP) by magnetic resonance neurography (MRN).

**Methods:**

Twenty patients (7 female, 13 male, 58.9±10.0 years) with mild to moderate OXA-PNP and 20 matched controls (8 female, 12 male, 55.7±15.6 years) were prospectively enrolled. All patients underwent a detailed neurophysiological examination prior to neuroimaging. A standardized imaging protocol at 3.0 Tesla included the lumbosacral plexus and both sciatic nerves and their branches using T2-weighted fat-saturated sequences and diffusion tensor imaging. Quantitative assessment included volumetry of the dorsal root ganglia (DRG), sciatic nerve normalized T2 (nT2) signal and caliber, and fractional anisotropy (FA), mean diffusivity (MD), axial (AD) and radial diffusivity (RD). Additional qualitative evaluation of sciatic, peroneal, and tibial nerves evaluated the presence, degree, and distribution of nerve lesions.

**Results:**

DRG hypertrophy in OXA-PNP patients (207.3±47.7mm^3^ vs. 153.0±47.1mm^3^ in controls, p = 0.001) was found as significant morphological correlate of the sensory neuronopathy. In contrast, peripheral nerves only exhibited minor morphological alterations qualitatively. Quantitatively, sciatic nerve caliber (27.3±6.7mm^2^ vs. 27.4±7.4mm^2^, p = 0.80) and nT2 signal were not significantly changed in patients (1.32±0.22 vs. 1.22±0.26, p = 0.16). AD, RD, and MD showed a non-significant decrease in patients, while FA was unchanged.

**Conclusion:**

OXA-PNP manifests with morphological and functional correlates that can be detected *in vivo* by MRN. We report hypertrophy of the DRG that stands in contrast to experimental and postmortem studies. DRG volume should be further investigated as a biomarker in other sensory peripheral neuropathies and ganglionopathies.

## Introduction

Oxaliplatin (OXA) is a third-generation platinum derivative that is currently utilized as first-line adjuvant and palliative therapy in advanced colorectal cancer [1, 2] and is also increasingly applied in other forms of gastrointestinal malignancies.[3, 4] Used in combined regimens with other substances, OXA is associated with prolonged disease progression-free and overall survival.[5] OXA has a good tolerability profile except for a painful peripheral neuropathy (PNP) as principal adverse effect of the substance, which manifests itself in two forms. The acute form of cold-induced transient hyperexcitability phenomena occurs during or shortly after administration of the substance and is probably due to interaction with sodium voltage-gated channels.[6] This form is fully reversible and does not require discontinuation of treatment. The chronic form presents as a distal-symmetric sensory neuropathy with sensory loss, paresthesia, and dysesthesia, and is only incompletely reversible.[7]

The chronic oxaliplatin-induced peripheral neuropathy (OXA-PNP) is dose-dependent and occurs in milder forms (National Cancer Institute common toxicity criteria grade 1–2) in the majority of patients upon completion of OXA-containing regimen while severe forms (grade 3–4) are reported in 10–20% of patients.[8] It causes progressive long-term neurological deficits and serious functional difficulties in daily life, severely reducing quality of life.[9, 10] These deterministic adverse effects are dose-limiting, such that OXA-PNP is the main cause of dose reductions or discontinuation of OXA-based treatment and thus compromises therapeutic outcome.[8]

Chronic OXA-PNP is believed to mainly result from accumulation of platinum compounds in the dorsal root ganglia (DRG) of the peripheral nervous system (PNS), causing apoptosis and eventually neuronal atrophy.[11] Directly assessing the extent of damage to the PNS is difficult due to its anatomical dissemination. Electrophysiological methods can only indirectly and functionally investigate the DRG. The nerve portions that are better accessible to electrophysiology or even nerve biopsy such as the sural nerve may not be representative for the potential early changes of OXA-PNP in DRG and proximal nerve portions.

Deeper structures of the PNS such as the DRG, plexus, and proximal nerve portions can be investigated by MRI, or magnetic resonance neurography (MRN).[12, 13] MRN principally relies on T2-weighted sequences with high-resolution to sensitively detect and localize neural lesions.[14] Nerve lesions can further be very sensitively characterized using diffusion tensor imaging (DTI) which is based on the normally highly directional diffusion of water protons in peripheral nerves and which may be disrupted in damaged nerve portions by axonal loss, myelin sheath breakdown, and other factors [15]. In this study, we investigated early changes to the PNS *in vivo* using an MRN protocol with morphological and functional techniques in patients treated with OXA.

## Patients and methods

### Patients and treatment regimen

This study was approved by the ethics board of the medical faculty of Heidelberg University (S-357/2012), and written informed consent was obtained from all participants. Patients were eligible for the study if they had developed symptoms of a polyneuropathy after having received a cumulative oxaliplatin dose of at least 250 mg/m2 for various forms of gastrointestinal cancer (see Table 1 for detailed patient data). Exclusion criteria were the following: age < 18 or > 75; any history of symptomatic peripheral artery or cerebrovascular disease, alcoholism, end-stage renal disease, or any other disease known to be related to the manifestation of peripheral neuropathy (e.g., autoimmune disease, systemic vasculitis, or infectious diseases) except for chemotherapeutic treatment and diabetes mellitus; and any contraindication for MRI.

**Table 1:** Patient demographics and clinical data.

#	age	diagnosis	cumulative dose	time between last oxaliplatin administration and MRI	CTC grade	OxaPNP Grade	chemotherapy
			[mg/m^2^]	days			
1	67	rectal cancer, SM, P	969	36	2	3	6x FOLFOX-6, 6x FOLFOX-6/Bevacizumab
2	62	rectal cancer, SM, A	425	8	1	2	4x FOLFOX-4, 1x FOLFOX-6
3	77	gastric cancer, MM, P	1545	8	1	1	6x EOX, 9x FLO
4	57	gastric cancer, SM, P	595	111	1	3	7x FLOT, 6x FOLFIRI
5	61	gastro-esophageal junction cancer, SM, P	1445	8	1	1	9x FLOT, 8x FLO
6	58	rectal cancer, SM, P	765	90	2	3	9x FOLFOX-6, 3x FOLFIRI
7	47	colon cancer, A	816	7	2	3	10x FOLFOX-6
8	64	pancreatic cancer, SM, P	255	7	1	1	3x FOLFIRINOX
9	74	rectal cancer, MM, P	884	112	2	4	12x FOLFOX-6/Cetuximab, 6x FOLFIRI/Bevacizumab
10	45	pancreatic cancer, SS, P	510	7	1	1	6x FOLFIRINOX
11	53	pancreatic cancer SM, P	646	7	1	3	8x FOLFIRINOX
12	59	rectal cancer SM P	425	8	1	1	RCTX (5-FU), 1x FOLFOX-4, 1x FOLFOX-6/Cetuximab, 3x FOLFOX-6/Panitumumab
13	47	colon cancer, A	850	51	2	3	10x FOLFOX-6, 2x 5-FU
14	52	pancreatic cancer SM, P	986	40	2	3	12x FOLFIRINOX
15	63	colon cancer, SM, P	1153	7	2	4	6x FOLFIRI, FUFOX, 36x FOLFIRI, 2x FOLFIRI/Cetuximab, 6x FOLFIRI/Bevacizumab, 23x CAPIRI/Bevacizumab, 2x 5-FU(AIO)/Bevacizumab, 5x 5-FU(deGramont)/Bevacizumab, 5x FOLFOX-6, 2x FOLFOX-6/Panitumumab
16	62	rectosigmoid cancer, A	425	7	1	1	5x FOLFOX-6
17	58	rectal cancer, SM, P	1275	33	2	3	12x FOLFOX-6/Panitumumab, 1x 5-FU(deGramont)/Panitumumab, 6x FOLFOX-6/Panitumumab, 1x 5-FU(deGramont)/Panitumumab
18	70	pancreatic cancer, MM, P	527	13	1	1	RCTX (Gemcitabin), 4x FOLFIRINOX, 4x Gemcitabin/Erlotinib, 2x OFF
19	41	rectal cancer, SM, P	425	9	1	1	5x FOLFOX-6/Bevacizumab
20	71	pancreatic cancer, MM, P	476	8	2	3	6x Gemcitabin, 3x OFF

A: adjuvant, CAPIRI: capecitabine+irinotecan; FOLFIRI: 5-FU+folinic acid+irinotecan; 5-FU: 5-fluorouracil; MM: metachronous metastases; RCTX: radiochemotherapy; SM: synchronous metastases; see S1 Table for details concerning oxaliplatin-containing regimens (EOX, FLO, FLOT, FOLFIRINOX, FOLFOX-4, FOLFOX-6, FUFOX, OFF)

Patients were treated with OXA-containing regimens according to standard of care at the National Center for Tumor Diseases, Heidelberg University Hospital, Germany. Chemotherapy regimens were applied in a palliative, adjuvant and/or neoadjuvant intention. Patients were regularly seen in the outpatient clinic, and tumor assessment and dose adjustment for toxicities were performed accordingly. Detailed regimens are listed in S1 Table.

### Clinical and neurophysiological assessment of OXA-PNP

Patients were clinically assessed using the peripheral sensory neuropathy subscale of the National Cancer Institute (NCI) Common Toxicity Criteria for Adverse Events Scale (CTC-AE) Version 4.03 with the following grading system: Grade 1 (mild)—asymptomatic, loss of deep tendon reflexes or paresthesia; Grade 2 (moderate)—moderate symptoms limiting instrumental activities of daily living; Grade 3 (severe)—severe symptoms, limiting self care activities of daily living; and Grade 4—life threatening consequences, urgent intervention indicated. In addition, an OXA-specific grading system (OXA-PNP scale) modified according to Lévi was applied: Grade 1 ––paresthesia and/or dysesthesia with complete regression within 7 days; Grade 2 ––paresthesia and/or dysesthesia with complete regression within 14 days; Grade 3 ––paresthesia and/or dysesthesia with incomplete regression between courses; Grade 4 ––paresthesia and/or dysesthesia with functional impairment.[16]

Patients additionally underwent nerve conduction studies (NCS) of the right sural, right peroneal, and left tibial nerves by surface electrostimulation at standard sites (distal motor latencies, compound-motor or sensory nerve action potentials, motor and sensory nerve conduction velocities, F-wave latencies and persistencies). Skin temperature was controlled at a minimum of 32°C, and NCS cut-off values were adjusted for age. Moreover, each patient underwent somatosensory-evoked potentials of the tibial nerves, and a needle electromyography of the right anterior tibial muscle.

### MRI data acquisition

High resolution MRI examinations had large coverage over the lumbosacral plexus and both legs and were performed on a 3 Tesla MR scanner (TRIO, Siemens, Erlangen, Germany) by the following protocol of pulse sequences:

1. Lumbosacral plexus: 3D T2-weighted inversion recovery SPACE (Sampling Perfection with Application-optimized Contrasts using different flip angle Evolution) sequence with 100 axial reformations for imaging of the lumbosacral plexus and spinal nerves: repetition time/echo time (TR/TE) 3,000/202 ms, effective TE 68ms, time of inversion 210 ms, field of view (FOV) 305×305 mm^2^, matrix size 320×320×104, voxel size 1.0×1.0×1.0 mm^3^.
2. T2-w imaging of both legs: Axial T2-weighted turbo-spin-echo two-dimensional sequences with spectral fat saturation: TR/TE 5970/55 ms, FOV 150×150 mm^2^, matrix size 512×512, slice thickness 3.5 mm, interslice gap 0.35 mm, voxel size 0.4×0.3×3.5 mm^3^, and 45 slices. Two slabs per leg: one at mid-thigh level by alignment of the distal slab end with the patella, one at calf level by alignment of the proximal slab end with the tibiofemoral joint.
3. DTI of right and left thigh: Diffusion-weighted echo-planar-imaging sequence: TR/TE 3800/99 ms, b-value 0 and 800 s/mm2 (encoded in monopolar 19 directions), readout bandwidth 1395 Hz/px, 18 slices, interslice gap 1.2 mm, FoV 150x150 mm2, acquisition matrix 128x128, in-plane-resolution 1.17x1.17 mm^2^, slice thickness 4 mm, number of excitations = 2.

The net imaging time was 42 minutes (both legs per patient), with coil repositioning requiring an additional 15–20 minutes.

### Quantitative image analysis

#### DRG volumetry

DRG volumes were assessed on 3D images of the lumbosacral plexus by measuring the largest DRG diameters in coronal and axial reformations. Slice positions were chosen separately for L4, L5, and S1 nerve roots and for the left and right side each. DRG volume was estimated using the following approximation: volume in mm^3^ = horizontal x sagittal x coronal diameter / 2 (derived in analogy to the mathematical equation for the volume of an ellipsoid).

#### Peripheral nerve T2-weighted imaging

Sciatic nerves were assessed at thigh level for the sciatic nerve, and at calf level for peroneal and tibial nerves by manually drawing the precise circumference of the nerve on every aquired image slice for read-out of caliber and T2-weighted values of this region of interest (ROI). Values for nerves were divided by corresponding values for adjacent muscle (biceps femoris for sciatic nerve, soleus muscle for tibial and peroneal nerves) to yield a normalized nerve T2 signal: nT2 (nerve) = ROI T2 (nerve) / ROI T2 (muscle).

#### Diffusion tensor imaging

DTI postprocessing and quantitative analysis was performed using FSL Diffusion Toolbox (FDT) of the FMRIB free software library (FSL, http://fsl.fmrib.ox.ac.uk/fsl) v5.0.6 (Oxford, UK) as described previously.[15] Precise manual segmentations of the sciatic nerve divided into its peroneal and tibial nerve portion were performed on each image slice of the acquisition volume at b = 0 images. The diffusion tensor model was fitted on DWI images yielding maps of the first, second and third eigenvalue from which parameter maps of fractional anisotropy (FA), radial diffusivity (RD), axial diffusivity (AD), and mean diffusivity (MD = apparent diffusion coefficient, ADC) were calculated.

### Statistical analysis

All results are expressed as mean ± standard deviation. Values for nerve nT2 and caliber were obtained by averaging all individual slices, and separately for the right and left leg. Mean values of these parameters were compared between patient and control groups using a Mann-Whitney rank sum test with the significance level set to p<0.05.

Correlation analysis was performed by calculating Pearson’s correlation coefficient between DTI values and electrophysiological results. In tests where no electrophysiological response could be elicited in some patients, Spearman’s rho was calculated instead. Statistical analysis was performed in SigmaPlot 13.0 (Erkrath, Germany).

### Qualitative image analysis

T2-weighted images of the sciatic nerve at thigh level and its branches at calf-level were additionally evaluated qualitatively by two readers with more than 8 (PB) and 5 (DS) years of experience in MRN. Sciatic nerve as well as peroneal and tibial nerve appearance was graded in consensus with regard to

1. Presence of abnormally increased intraneural T2-w signal: present / not present
2. Severity of T2-w signal increase: none / weak / moderate / severe
3. Distribution of signal abnormalities within the nerve cross section as well as the longitudinal extension: none / partial nerve cross section with somatotopical order / partial nerve cross section in random fashion / entire nerve cross section.

## Results

### Clinical patient data and electrophysiological findings

Twenty patients (7 female, 13 male, 58.9±10.0 years) with oxaliplatin-induced peripheral polyneuropathy and 20 age-matched healthy control subjects (8 female, 12 male, 55.7±15.6 years) were prospectively enrolled. The average cumulative OXA dose was 770±367 mg/m^2^ (range 255–1545 mg/m^2^). Mean time period of last OXA administration before MRI was 29±35 days (range 7–112 days). Mean CTC grade was 1.45±0.51 (range 1–2) and mean OXA-PNP grade was 2.2.5±1.12 (range 1–4). Individual patient data including severity of neuropathy are presented in Table 1. By electrophysiological examination, most patients had mild signs of axonal neuropathy (detailed results in Table 2).

**Table 2:** Electrophysiological test results.

ID	age	summary	f-wave-latency and persistence				motor NCV, latency, and MSAP						sensory NCV and SNAP		SSEP latency and amplitude			
			right peroneal		right tibial		right peroneal			right tibial			right sural		right tibial		left tibial	
			(ms)	%	(ms)	%	(m/s)	(ms)	(uV)	(m/s)	(ms)	(uV)	(m/s)	(μV)	P40 (ms)	N35-P40 (μV)	P40 (ms)	N35-P40 (μV)
1	67	axonal sensory polyneuropathy	51.7	67	51.8	100	40	4.3	6	43	5	16	n.p.		58.0	0.5	60.0	0.6
2	62	possible sensory axonal polyneuropathy	46.1	30	44.2	100	44	3.6	7.6	51	4	19	48	13	43.8	0.7	38.9	0.9
3	77	demyelinating sensory polyneuropathy of unclear origin	47.0	>90	44.0	>90	49	4.6	6	43	4.1	16	29	7	44.2	1.9	49.1	1.5
4	57	minor axonal sensory-motor polyneuropathy	53.0	>90	53.0	>90	43	4.1	4.7	n.p.	4.7	21	46	2	44.4	0.6	43.4	0.4
5	61	no signs of polyneuropathy	52.8	>90	53.4	54	49	6.0	3.7	43	5	15	43	20	41.4	0.7	43.1	0.5
6	58	minor axonal mainly sensory polyneuropathy	n.p.		48.0	100	45	3.8	5.6	51	3.4	23	41	6	n.p.			
7	47	minor axonal-demyelinating sensory polyneuropathy	-	-	-	-	44	4.3	12.5	47	4.6	16	33	10	41.4	0.2	43.9	0.5
8	64	minor axonal sensory-motor polyneuropathy	54.2	>90	53.9	>90	45	4.6	2.8	58	3.9	24	37	8	40.1	1.0	40.8	0.9
9	74	axonal. mainly sensory polyneuropathy	n.p.		55.0	>90	38	4	3	41	4.7	7	n.p.		63.5	0.2	60.3	0.3
10	45	possible. beginning sensory polyneuropathy	44.4	>90	43.4	>90	48	4.9	11	54	5.1	27	36	21	37.8	2.0	39.2	2.3
11	53	axonal sensory-motor polyneuropathy	n.p.		57.0	>90	-	n.p.	-	44	3.8	25	41	10	48.7	1.4	51.8	0.4
12	59	declined examination	-	-	-	-	-	-	-	-	-	-	-	-	-	-	-	-
13	47	axonal-demyelinating sensory polyneuropathy	49.4	>90	51.3	>90	47	4.8	5.4	44	3.9	29	30	5	56.7	1.3	56.7	2.0
14	52	axonal sensory polyneuropathy	59.0	>90	56.0	>90	42	4.4	5.3	46	5.3	13	n.p.		n.p.			
15	63	declined examination	-	-	-	-	-	-	-	-	-	-	-	-	-	-	-	-
16	62	minor f-wave abnormality. no certain sign of polyneuropathy	49.8	50%	51.0	>90	48	3.9	8.6	44	4.0	21	44	12	n.p.			
17	58	declined examination	-	-	-	-	-	-	-	-	-	-	-	-	-	-	-	-
18	70	no certain sign of polyneuropathy	53.7	>90	55.0	>90	44	4.4	9	42	5.1	15	55	19	46.6	2.6	46.4	1.7
19	41	minor sensory axonal polyneuropathy	57.3	>90	62.2	>90	43	3.9	5	44	5.1	17	37	4	46.0	3.4	46.3	2.6
20	71	sensory axonal polyneuropathy	46.9	>90	54.2	>90	41	4.5	3.6	40	5.7	12	40	1	45.2	0.8	46.4	1.2

CMAP—compound motor action potential; dmL—distal motor latency; NCV—nerve conduction velocity; SNAP—sensory nerve action potential; SSEP—somatosensory evoked potential. n.p.—no reproducible potential. Patient 12, 15, and 17 declined to undergo the examination for fear of pain. Patient 7 interrupted the examination before F-wave diagnostics were determined.

### Imaging findings

#### Spinal ganglia

**Fig 1:**
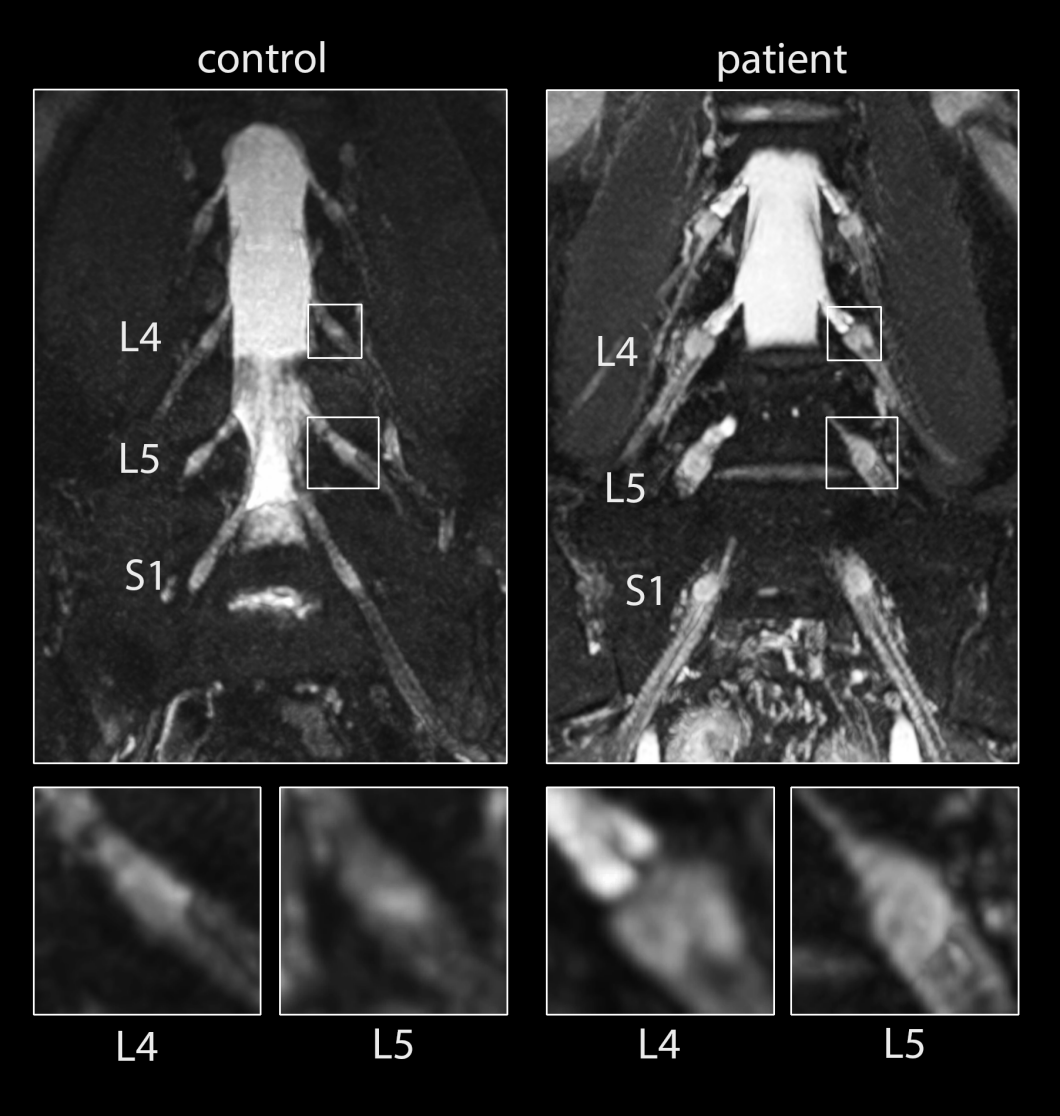
Lumbosacral plexus with dorsal root ganglia. Images were obtained from maximum intensity projections of spinal cord and lumbosacral plexus images acquired by a SPACE STIR sequence. Left column shows images from a healthy control with small, normal sized dorsal root ganglia. Right column shows images from a patient after exposure to a cumulative dose of 510 mg/m^2^ OXA. Insets in lower line show magnifications of L4 and L5 DRG. Nerve roots filled with cerebrospinal fluid contact the DRG, whose fibers then continue into the spinal nerve roots.

Significant DRG hypertrophy was observed in OXA-treated patients (Fig 1). For L4, DRG volume in patients was 206.1±68.2mm^3^ vs. 132.1±56.8mm^3^ in controls (p<0.001), for L5 volumes were 187.8±46.8mm^3^ vs. 150.5±47.9mm^3^ (p = 0.017), and for S1 238.4±55.0mm^3^ vs. 176.2±65.5mm^3^ (p<0.001). This corresponded to a mean DRG volume increase of 25% (L5) to 56% (L4) in patients. Averaging all three levels of evaluated DRG, volume in patients was increased to 210.8±47.6mm^3^ vs. 156.5±45.0mm^3^ (p<0.001).

#### Sciatic nerve and branches

**Fig 2:**
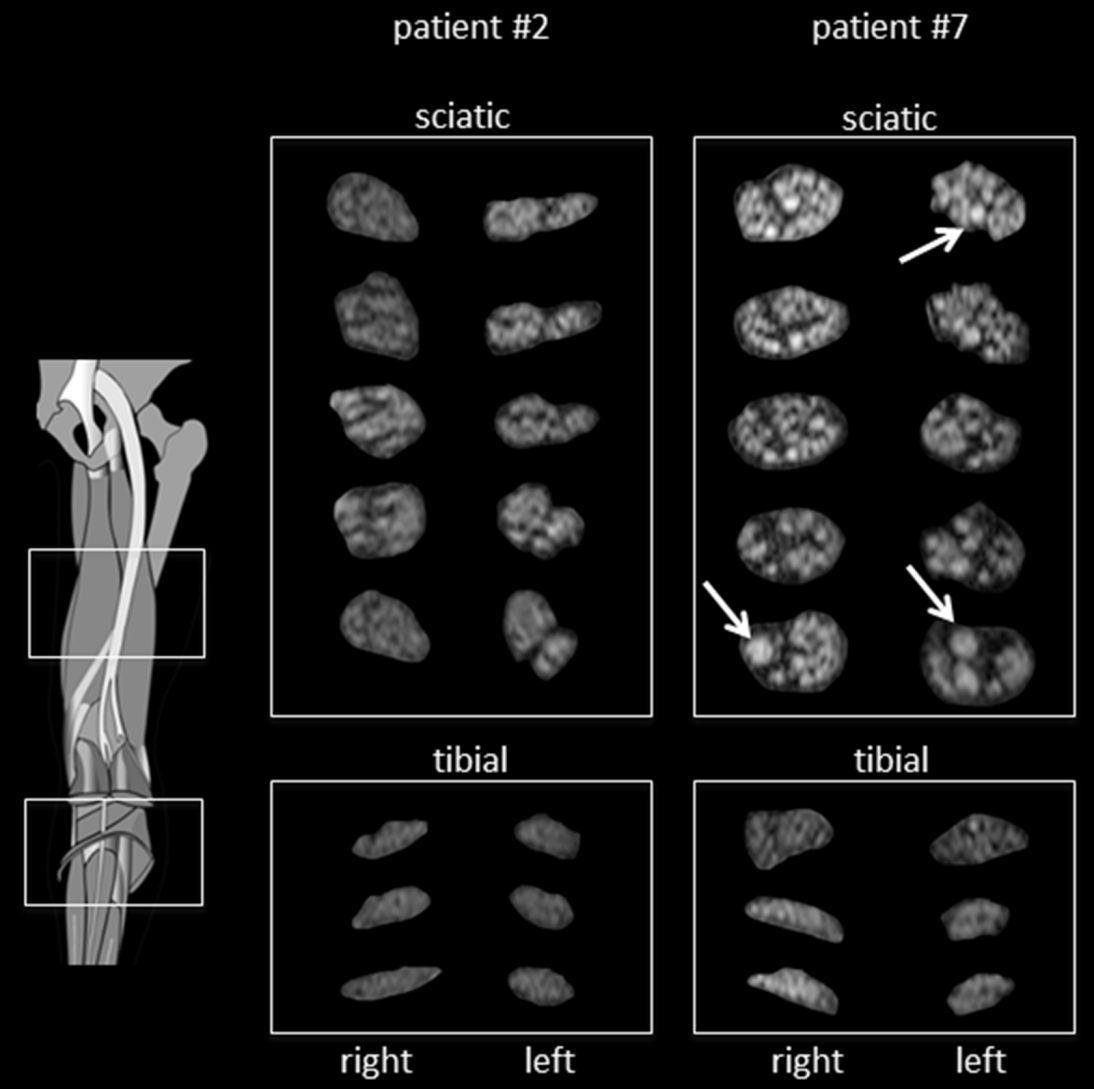
Nerve appearance in T2-w at thigh and calf level. Exemplary OXA-treated patients without apparent peripheral nerve alterations (patient #2, left column) and with apparent minor nerve alterations (patient #7, right column). The sciatic nerves on the right exhibit slightly increased T2-w signal and caliber thickening of individual fascicles (arrow). Overall, peripheral nerve changes are mild.

The sciatic, peroneal and tibial nerves were imaged on both sides. Peripheral nerves in OXA-PNP patients exhibited heterogeneous signal changes (Fig 2). In 7 of 20 patients, no T2-w signal alterations were observed. In the other 13 patients, T2-w signal alterations were rated as ‘weak’ in 6 and ‘moderate’ in 7. In 11 of these 13 patients, lesions were scattered in a random order both in the nerve cross section as well as in longitudinal extension, while the entire nerve cross section was affected in 2. In patients with nerve lesions, mainly the sciatic nerves appeared affected while peroneal and tibial nerves exhibited relatively few lesions. In clinically healthy controls, 5 out of 20 had nerve T2-w signal alterations, rated as weak in 4 and moderate in 1, all with random distribution.

Overall, normalized sciatic nerve T2 signal was slightly but not significantly increased in patients (1.32±0.22 vs. 1.22±0.26, p = 0.16). Likewise, sciatic nerve caliber was unchanged compared to controls (27.3±6.7mm^2^ vs. 27.4±7.4mm^2^, p = 0.80).

#### Diffusion tensor imaging

**Fig 3:**
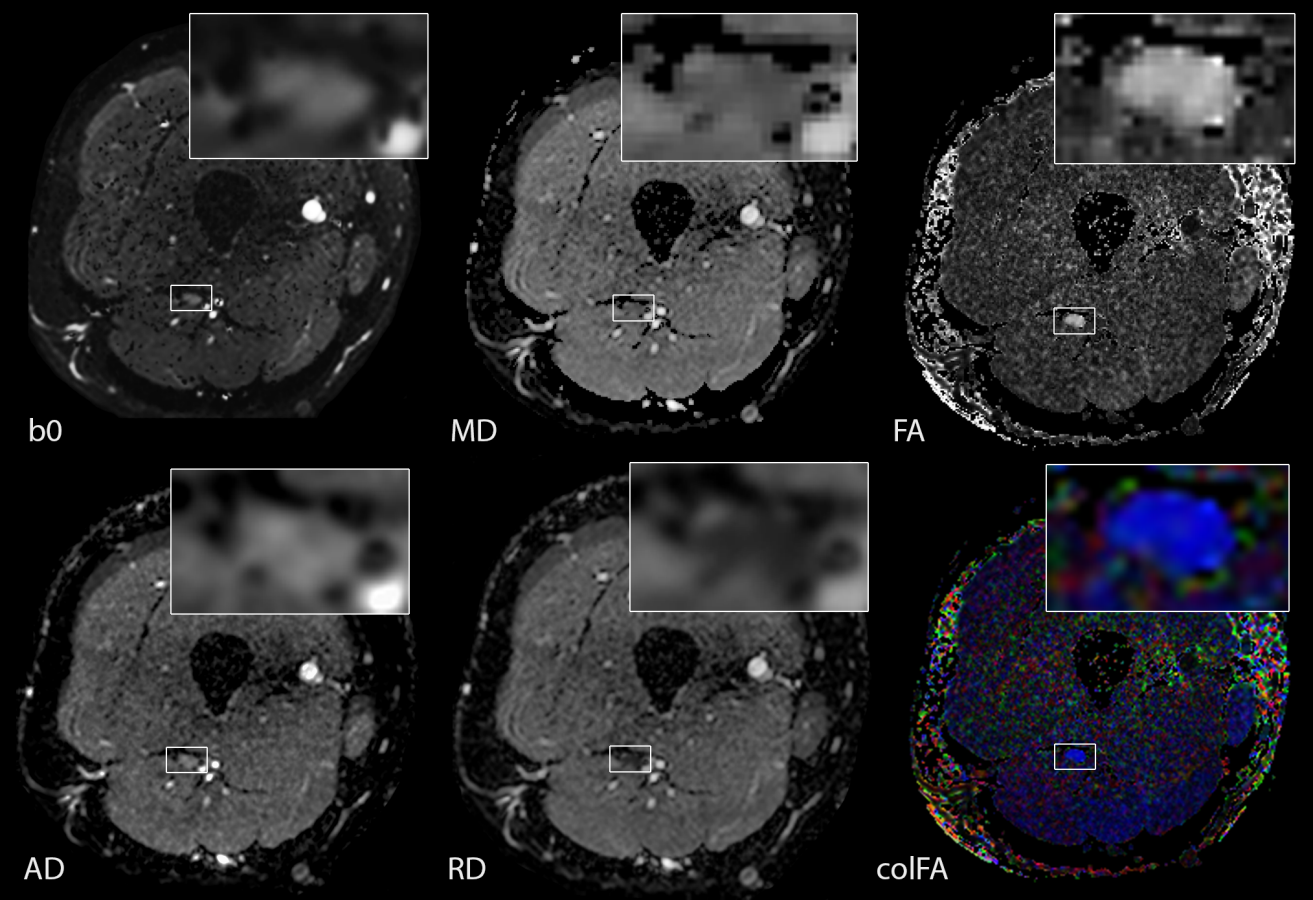
DTI nerve appearance at thigh level. The six images demonstrate DTI images of an exemplary OXA-treated patient. B0 maps were used for nerve identification and segmentation. MD, AD, and RD show parameter maps. FA maps are shown in grey-scale, and additionally color-maps for indication of preferential diffusion direction.

**Fig 4:**
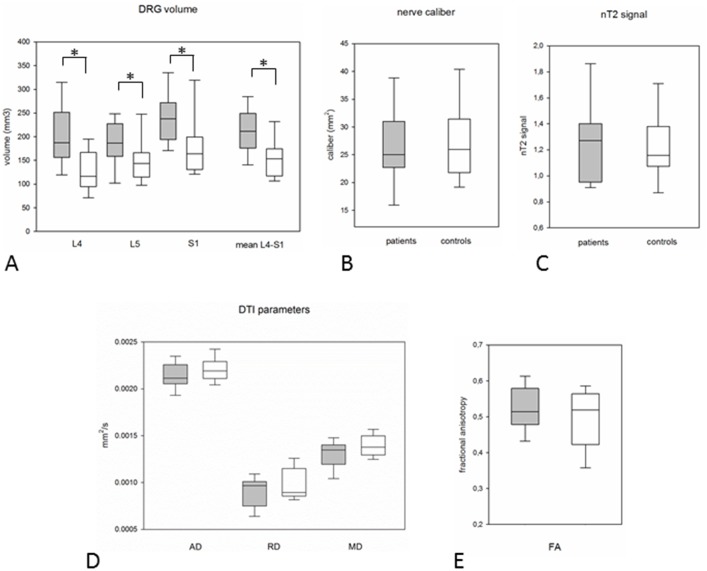
Boxplots of quantitative results. [A] shows values for DRG in OXA-treated patients (grey boxes) vs. controls (white boxes). [B] shows normalized T2 values for the sciatic nerve at mid-thigh level. [C] gives values for the sciatic nerve caliber at mid-thigh level. [D] shows values for DTI parameters AD, RD, and MD while [E] depicts resulting FA values. Boxplots give median, 25% and 75% quartile values. Whiskers indicate 5% and 95% percentile.

DTI of peripheral nerves was assessed by four read-out parameters: FA, AD, RD, and MD (Fig 3). The most commonly used marker for nerve integrity, FA, did not differ between patients and controls (0.52±0.07 vs. 0.50±0.09, p = 0.53). AD (2.11±0.22 vs 2.21±0.14 x10^-3^ mm^2^/s, p = 0.21), RD (0.90±0.16 vs 0.98±0.17 x10^-3^ mm^2^/s, p = 0.16), and MD (1.31±0.16 vs 1.40±0.12 x10^-3^ mm^2^/s, p = 0.13) were all non-significantly decreased in patients (Fig 4). No side difference was noted in patients.

#### Correlation analyses

As a test whether read-out parameters of DTI as a functional imaging technique actually represented nerve function, DTI values were compared with electrophysiological test results. Tibial nerve N35-P45 amplitude correlated in a negative inverse linear manner with tibial nerve AD (r = -0.47, p = 0.05), MD (r = -0.60, p = 0.01), and RD (r = -0.57, p = 0.02). Tibial nerve AD also correlated with F-wave latency (Spearman’s rho = -0.64, p = 0.006). FA did not correlate to any electrophysiological parameter.

None of the electrophysiological results correlated to DRG size. Further, dose did not correlate with DRG volume in our sample nor with any of the other imaging read-out parameters. Also, timing of the MRI after last administration of oxaliplatin did not result in significant changes in any of the assessed morphological and functional parameters. Individual values are provided in S2 Table.

## Discussion

The current study presents *in vivo* morphological and functional imaging results in patients with early OXA-PNP and identifies dorsal root ganglia hypertrophy as a hitherto unreported finding in OXA-PNP.

Direct investigation of damage to the peripheral nervous system by OXA has so far been precluded by the difficult accessibility and the anatomical dissemination of the PNS elements. Assessment of nerve damage has previously relied on neurological examinations, electrophysiology, and skin biopsy, all of which have limitations in accurately assessing the extent of damage to the PNS. For example, scoring systems based solely on neurological examinations have performed poorly in reliably assessing the severity of peripheral neuropathy.[17, 18] Multiple variants of electrophysiological techniques have attempted to quantify peripheral nerve damage by OXA. Two extensive studies on OXA-treated patients have indeed shown changes in electrophysiological parameters and have identified risk factors for developing more severe forms of OXA-PNP.[19, 20] While electrophysiological methods are excellent tools to study the function of peripheral nerves, they do not provide any information beyond the electrical function of the largest myelinated nerve fibers and, further, reliably access mainly the distal nerve portions, such as the peroneal, tibial or sural nerve. Intraepidermal nerve fiber density measures acquired by skin punch biopsies or sural nerve biopsy are one method that can be used both in animal models and patients.[21] It is, however, invasive and only measures small distal nerve branches in a single skin area (commonly obtained from the lateral lower leg) and gives no direct measures of larger nerve fiber integrity or DRG. To our knowledge, imaging biomarkers in OXA-PNP have not been described previously.

Based on electrophysiological evidence in man and experimental animal studies, the damage of OXA to the PNS is believed to occur mainly at the DRG with ensuing sensory neuronopathy of myelinated sensory nerve fibers. To our knowledge, no histopathological studies exist on the effects of OXA on the whole of the PNS in patients. Two single postmortem study after cisplatin administration found platinum levels which correlated linearly with the exposure to antemortem platinum-derived medication.[22, 23] These were highest in the DRG and to a lesser degree detected in peripheral nerves. Experimental animal studies also describe the DRG as the main localization of nerve damage. However, these *ex-vivo* studies report hypotrophy of the DRG along with neuronal atrophy of the nucleus and soma, [22–27] which appears contrary to the hypertrophy found on MRI. This apparent discrepancy might be explained by different time points of examination since MRI dates in our patients were later than the time of harvesting neural tissue in animals and earlier than human autopsy studies.

OXA-induced damage seems relatively restricted to the DRG because more distal parts of the PNS, namely the sciatic, peroneal and tibial nerves did not generally show conspicuous lesions in all patients. Peripheral nerve findings in experimental neuroanatomical and electrophysiological studies have principally been axonopathy with largely intact myelin sheaths and endoneural space, and no alteration in size and number of endoneural vessels.[25, 26] Electrophysiological evidence and rare sural nerve biopsies in patients have been consistent with these experimental results. Also consistent are our MRN findings of scattered weak T2-w changes with normal caliber which are minor compared to previously investigated polyneuropathies.[12, 13] Further, DTI values which have been shown to be highly sensitive to even slight nerve integrity alterations [15, 28] were only slightly and not significantly altered in our population. The minor changes we did observe in nT2 and DTI values may have been due to subacute axonal degeneration of few nerve fibers and little myelin sheath damage which is in line with our electrophysiological results. Overall, neither normalized signal intensity of T2-w-based MRN nor DTI of peripheral nerves can be considered a useful biomarker in OXA-PNP.

DRG size did not correlate significantly with dose nor with any of the electrophysiological measures obtained here. We believe that the reason for this absence of strong correlations in this study is the high inter-individual variation of DRG size. A measure to counteract this high variability in future studies might be to normalize DRG size to baseline values before chemotherapy.

In previous patient investigations, little attention has been paid to the DRG although they can be accurately identified and localized by MRI.[29, 30] Only one MRI-based study focused on DRG in peripheral neuropathy and found hypertrophy in 5 of 10 patients with Sjögren’s syndrome.[31] This limited number of previous studies supports our finding of hypertrophy instead of the experimentally and postmortem reported hypotrophy.

More generally, the DRG is a prominent and frequent site for nerve damage due to its intense vascular supply and the local high-permeability of its blood-nerve barrier.[32, 33] This leaky capillary permeability is likely the route of access for many chemotoxic agents. Dynamic contrast-enhanced perfusion is an MRI-based technique to investigate the perfusion of DRG *in vivo* and may become a useful tool in the investigation of sensory peripheral neuropathies.[34]

For the prevention of OXA-PNP, a number of potential neuro-protective substances have been under development and investigation.[35, 36] However, until now, sufficient evidence for application of these substances into routine clinical practice could not be provided. A major difficulty in assessing efficacy of neuroprotective measures has been the lack of reliable tests to quantify nerve damage. Clinical grading systems such as the OXA-PNP scale used in this study have been shown to be more sensitive for progression of symptoms compared to standard CTC [16]. Additionally, self-questionnaires [37] may be used since comparison of physician-assessed and patient-reported outcome measures in PNP has shown discrepancies.[38] In terms of objective tests, electrophysiological parameters have been identified to predict patients at risk to develop OXA-PNP [19, 20] and may also serve as good indicators of disease severity, but their use in monitoring disease for nerve protection is unclear. MRI parameters might be employed as a useful adjunct for such studies.

Our study has several limitations. First, patients also received other chemotherapeutic agents whose effect on the PNS could not be controlled for. Second, our sample size was relatively small, although still sufficient to arrive at significant results. More subtle effects not described here may be found in larger trials. Third, the applied protocol is long and uncomfortable for patients, but since the main effect was found in the DRG and not in peripheral nerves, future studies can significantly shorten the protocol. And fourth, the correlation of imaging parameters with detailed questionnaires and advanced electrophysiological techniques such as quantitative sensory testing and laser-evoked potentials were not assessed here but should be investigated in the future.

In conclusion, this study offers an imaging analysis of OXA-PNP with the most prominent finding of DRG hypertrophy. Future studies with longitudinal design may investigate the development of DRG morphology and potentially additional functional parameters in OXA-PNP. The DRG will also be an interesting analysis target for other sensory neuropathies and ganglionopathies.

## Supporting information

S1 Table OXA-containing chemotherapy protocols. (TIF)

S2 Table Imaging data. Units are for DRG mm^3^, for nerve area (= caliber) mm^2^, and mm^2^/s for AD, RD, and MD. FA and nT2 are dimensionless. n.a.–not available. SD–standard deviation.
(TIF)
