# Supplementary figures and images for: Dorsal root ganglia hypertrophy as in vivo correlate of oxaliplatin-induced polyneuropathy
Source: PLoS One. 2017 Aug 24;12(8):e0183845. doi: 10.1371/journal.pone.0183845 (PMC5570356; doi:10.1371/journal.pone.0183845)

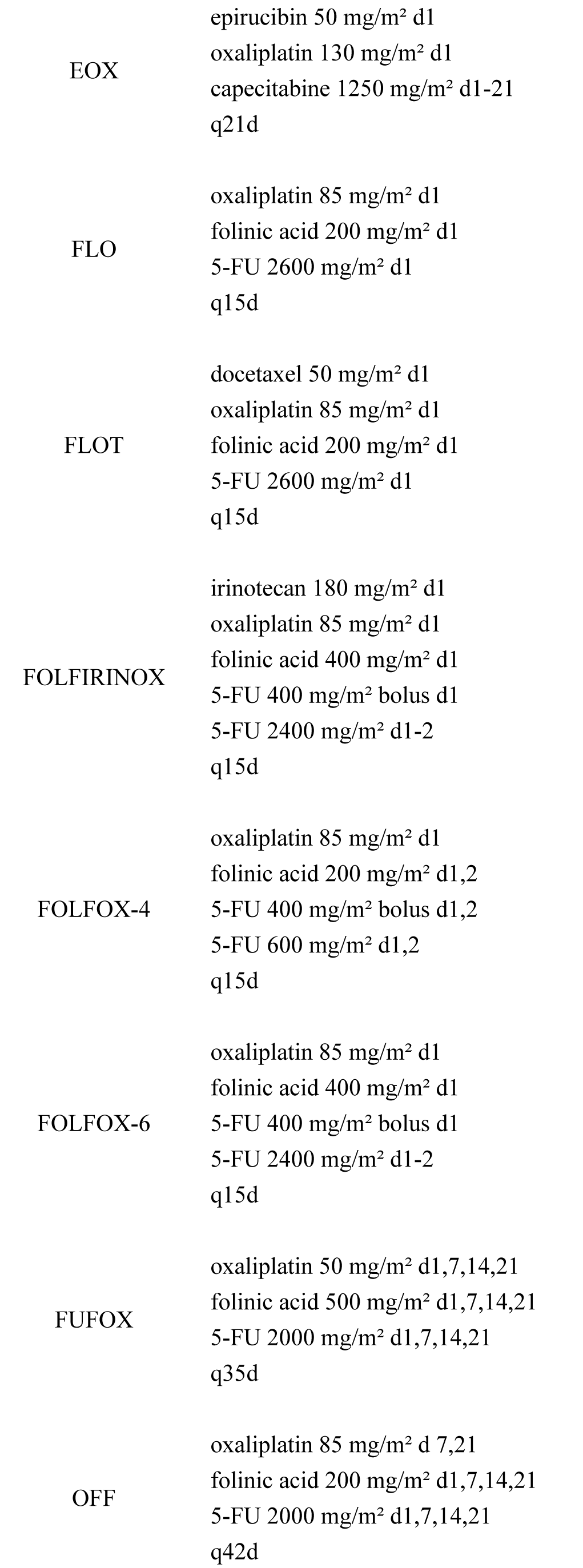

Supplement: S1 Table — (TIF) [file pone.0183845.s001.tif]

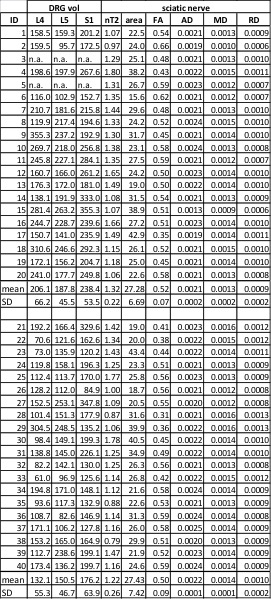

Supplement: S2 Table — Units are for DRG mm3, for nerve area (= caliber) mm2, and mm2/s for AD, RD, and MD. FA and nT2 are dimensionless. n.a.–not available. SD–standard deviation. (TIF) [file pone.0183845.s002.tif]
